# Estradiol-Estrogen Receptor α Mediates the Expression of the *CXXC5* Gene through the Estrogen Response Element-Dependent Signaling Pathway

**DOI:** 10.1038/srep37808

**Published:** 2016-11-25

**Authors:** Pelin Yaşar, Gamze Ayaz, Mesut Muyan

**Affiliations:** 1 Department of Biological Sciences, Middle East Technical University, Ankara 06800, Turkey

## Abstract

17β-estradiol (E2), the primary circulating estrogen hormone, mediates physiological and pathophysiological functions of breast tissue mainly through estrogen receptor α (ERα). Upon binding to E2, ERα modulates the expression of target genes involved in the regulation of cellular proliferation primarily through interactions with specific DNA sequences, estrogen response elements (EREs). Our previous microarray results suggested that E2-ERα modulates *CXXC5* expression. Because of the presence of a zinc-finger CXXC domain (ZF-CXXC), CXXC5 is considered to be a member of the ZF-CXXC family, which binds to non-methylated CpG dinucleotides. Although studies are limited, CXXC5 appears to participate as a transcription factor, co-regulator and/or epigenetic factor in the regulation of cellular events induced by various signaling pathways. However, how signaling pathways mediate the expression of *CXXC5* is yet unclear. Due to the importance of E2-ERα signaling in breast tissue, changes in the CXXC5 transcription/synthesis could participate in E2-mediated cellular events as well. To address these issues, we initially examined the mechanism whereby E2-ERα regulates *CXXC5* expression. We show here that *CXXC5* is an E2-ERα responsive gene regulated by the interaction of E2-ERα with an ERE present at a region upstream of the initial translation codon of the gene.

Estrogen hormones, particularly 17β-estradiol (E2) as the major estrogenic hormone in the circulation, play critical roles in the homeodynamic regulation of many organ and tissue, including breast tissue, functions. E2 is also involved in the initiation and development of target tissue malignancies. E2 signaling is mediated primarily by Estrogen receptor (ER) α and β, which are ligand-dependent transcription factors^1^,^2^. ERs are distinct gene products expressed in the same as well as different tissues at varying levels^1^,^2^. ERα is the major transcript expressed in breast tissue^1^,^2^.

ERα immediately after synthesis dimerizes and translocates predominantly to the nucleus independent of E2^3^. E2 binding leads to a conformational change in the receptor^4^. This structural alteration generates binding surfaces for effective interactions with co-regulatory proteins, enhances the stability and the association with DNA of the ERα dimer^2^,^3^,^4^,^5^,^6^,^7^. The nuclear E2-bound ERα regulates gene transcriptions through estrogen response element (ERE)-dependent and ERE-independent pathways^2^,^8^. EREs are permutations of the 5′-GGTCAnnnTGACC-3′ DNA palindrome, wherein ‘n’ denotes a non-specific three nucleotide spacer, located at various regions of gene loci^2^,^8^. The regulation of gene expressions by a direct interaction of E2-ERα with EREs is referred to as the ERE-dependent signaling pathway. Whereas, the transcriptional modulation of target genes through interaction of E2-ERα with transcription factors bound to their cognate regulatory elements on DNA denotes the ERE-independent signaling pathway^2^,^8^. The ERE-independent signaling participates in the fine-tuning of cellular responses; however, gene expressions mediated by E2-ER through the ERE-dependent signaling route are required for phenotypic changes in cell models^9^,^10^.

In a study using a microarray approach, we previously observed that *CXXC5* is an estrogen responsive gene regulated by ERα^10^. CXXC5, also referred to as the CXXC Finger Protein 5 (CF5), Retinoid-Inducible Nuclear Factor (RINF) or WT1-Induced Inhibitor of Dishevelled (WID), is considered to be a member of the CXXC-type zinc finger (ZF) protein family^11^,^12^,^13^,^14^. ZF-CXXC family includes CFP1 (CXXC protein finger 1, CXXC1), MBD1 (Methyl-CpG Binding Domain Protein 1, CXXC3), KDM2A & 2B (Lysine (K)-Specific Demethylase 2A & 2B; CXXC8 & CXXC2), DNMT1 (DNA (Cytosine-5-)-Methyltransferase 1, CXXC9) and TET1, 2, and 3 (Tet Methylcytosine Dioxygenase 1, 2, 3) proteins^15^. ZF-CXXC family proteins specifically recognize and bind to non-methylated CpG containing DNA, concentrated in regions known as CpG islands^15^. This binding is suggested to prevent DNA methylation leading to a state permissive to transcription^15^.

Structural and functional studies on CXXC5 are limited. However, it appears that *CXXC5* expressed in different tissues at varying levels^12^ is involved in the modulation of cellular proliferation, differentiation and death as a transcription factor, transcription co-regulator and/or chromatin modifier in response to retinoic acid, bone morphogenetic protein 4 (BMP4), Wnt signaling as well as to hypoxia^12^,^13^,^14^,^16^,^17^. The de-regulated expression of CXXC5 is also suggested to correlate with the development, and resistance to therapies, of various pathologies including cardiovascular disease, diminished ovarian reserve (DOR), Blepharophimosis Ptosis Epicantus inversus Syndrome (BPES), Acute Myeloid Leukemia (AML) and breast cancer^12^,^18^,^19^,^20^,^21^,^22^,^23^.

Because of the critical role of the E2-ERα signaling in physiology and pathophysiology of breast tissue, changes in the CXXC5 transcription/synthesis in response to E2 could participate in cellular events mediated by E2-ERα as well. To begin in addressing these issues, we initially examined the underlying mechanism by which E2-ERα signaling regulates the *CXXC5* gene expression using *in vitro* and *in cellula* approaches. Our results indicate that *CXXC5* is a *bona fide* E2-ERα responsive gene such that E2-ERα regulates the expression of *CXXC5* through a direct interaction with an ERE sequence present at a region upstream of the initial translation codon of *CXXC5*.

## Results

### Effects of E2 on transcript and protein levels of *CXXC5*

**Figure 1:**
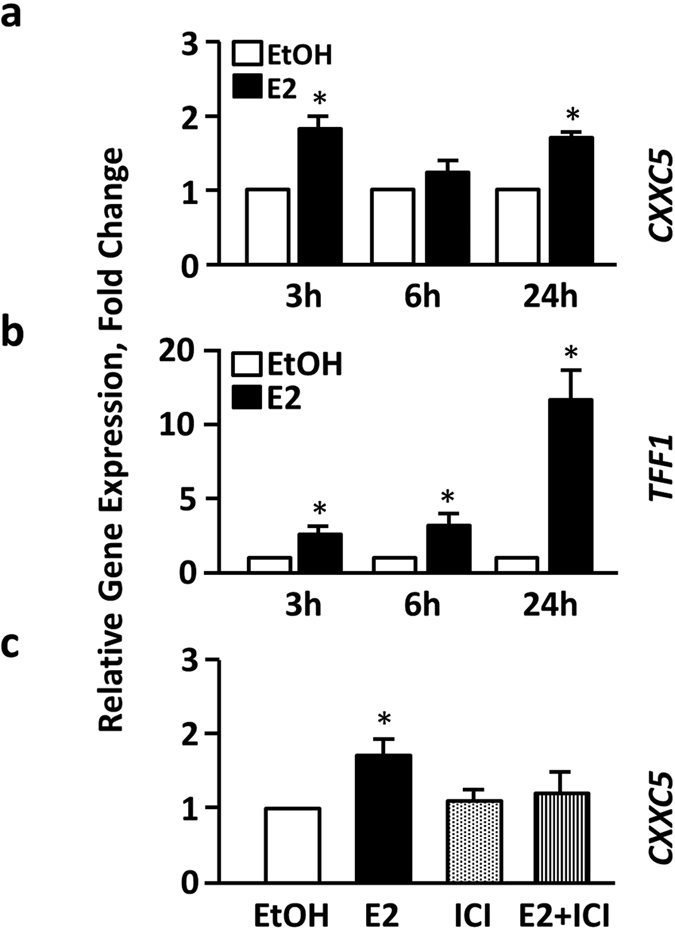
Transcriptional Responses of *CXXC5* and *TFF1* to ER ligands. MCF7 cells grown in medium containing charcoal-dextran treated fetal bovine serum (CD-FBS) for 48 h were treated without (ethanol, EtOH, 0.01% as vehicle control) or with 10^−9^ M E2 for 3 h, 6 h or 24 h. Cells were subsequently subjected to total RNA isolation for the expression of *CXXC5* (**a**) or *TFF1* (**b**). MCF7 cells were also treated without (EtOH) or with 10^−9^ M E2 and/or 10^−7^ M ICI for 24 hours for *CXXC5* expression (**c**). RT-qPCR results, which are the mean ± SD of three independent determinations in triplicates normalized to the expression of *RPLP0*, depict fold changes in mRNA levels in response to ligand compared with those treated with EtOH, which is set to 1 at each time point (**a**,**b**) or at 24 h (**c**). Asterisk (*) indicates significant change.

Our previous studies using microarrays suggest that *CXXC5* is an E2-ERα responsive gene^10^. We therefore wanted to verify with RT-qPCR that the expression of *CXXC5* is indeed mediated by E2 signaling in cells synthesizing ERα. MCF7 cells, derived from a breast adenocarcinoma, is an E2 responsive and ERα synthesizing cell line that is extensively used as a model for ERα-positive breast cancers^24^. To assess the expression of *CXXC5* in response to E2, MCF7 cells were cultured in charcoal-dextran treated fetal bovine serum (CD-FBS) to reduce/ablate steroid hormone levels for 48 h. Cells were then treated in the absence (0.01% ethanol control, EtOH) or the presence of 10^−9^ M E2, a physiological concentration, for 3 h, 6 h or 24 h. Samples were subsequently subjected to total RNA extractions and RT-qPCRs. Results were normalized by using the expression of *RPLP0*, which is one of the most reliable reference genes for normalization of RT-qPCR results in breast carcinomas^25^. We found that E2 augments the transcription of *CXXC5* in MCF7 cells in a biphasic fashion: E2 enhanced the transcription of *CXXC5* at 3 h and 24 h but was without an effect at 6 h (Fig. 1a). E2 on the other hand, induced the expression of *TFF1/pS2*, which is a well characterized E2-ERα responsive gene^26^,^27^ and used here as a positive control, at time points tested (Fig. 1b). The effects of E2 on *CXXC5* expression is ERα dependent; this is because the ER antagonist Imperial Chemical Industries 182,780 (ICI) at 10^−7^ M effectively prevented E2-mediated augmentation of the transcription of *CXXC5* (shown at 24 h in Fig. 1c) or *TFF1* (data not shown). These, together with the observations that E2 and/or ICI had no effect on the expression of *CXXC5* in ERα-negative MDAMB231 cells derived from a breast adenocarcinoma (data not shown), collectively suggest that the expression of the *CXXC5* gene in MCF7 cells is E2- and ERα-dependent.

**Figure 2:**
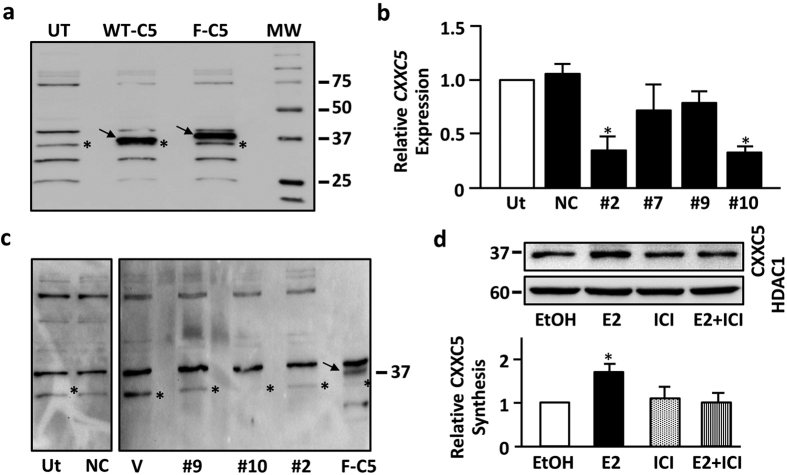
Detection of CXXC5 protein in MCF7 cells. (**a**) MCF7 cells were un-transfected (UT) or transfected with pcDNA3.1 (−) bearing WT-CXXC5 (WT-C5) or Flag-CXXC5 (F-C5) cDNA for 24 h. Cells were then subjected to nuclear protein extraction, SDS 10%-PAGE and WB using a CXXC5-specific antibody, ab106533. Star in the un-transfected (UT) lane denotes the putative endogenous CXXC5 protein, while arrows indicate the overexpressed WT-CXXC5 or Flag-CXXC. Molecular weight marker is in kDa. A representative image from two independent experiments is shown. It should be noted that we used 100 μg nuclear extracts of UT cells, while 25 μg of nuclear extracts of WT-C5 or F-C5 cells were used to prevent overshadowing effects of the overexpressed CXXC5 (Supplemental Data, Fig. 1). (**b**) MCF7 cells were transfected without (un-transfected, UT) or with AllStars negative control siRNA (NC), siRNA#2, #7, #9 or #10. 24 h later, cells were subjected to total RNA extractions and RT-qPCR using CXXC5 specific-primers. CXXC5 transcript levels in siRNA transfected cells were compared to levels in un-transfected cells, which was set to 1. Results are the mean ± SD of three biological repeats with three technical replicates. Asterisk (*) denotes significant change. (**c**) MCF7 cells were transfected for 24 h without (un-transfected, UT) or with AllStars (NC), siRNA#2, #7, #9 or #10. We also transfected cells with pcDNA3.1 bearing none (Vector, V) or WT-CXXC5 cDNA as control. 100 μg nuclear protein extracts, with the exception of F-C5 which was 25 μg to prevent the shadowing effect of the overexpressed protein on the endogenous protein, were subjected to WB using ab106533. Star denotes the endogenous CXXC5, while the arrow indicates the overexpressed Flag-CXXC5. A representative image from two independent experiments is shown. (**d**) Effects of ER ligands on endogenous CXXC5. MCF7 cells grown in medium containing CD-FBS for 48 h were treated without (EtOH, 0.01%) or with 10^−9^ M E2 and/or 10^−7^ M ICI for 24 hours. Nuclear extracts were subjected to WB using ab106533 or an HDAC1 antibody. A representative image from two independent experiments is shown. Changes in protein levels were quantified with ImageJ image processing program. Asterisk (*) denotes significant change.

To examine whether the enhanced *CXXC5* expression in response to E2 is reflected in the amount of CXXC5 protein, we used WB. *CXXC5* encodes a 322 amino-acid long protein with an estimated molecular weight of 33 kDa^11^,^12^. To study endogenous protein synthesis in MCF7 cells, we used commercially available, albeit limited number, of antibodies for CXXC5 in WB. Of the antibodies, only ab106533 detected a protein primarily in the nuclear fraction of MCF7 cells migrating at an estimated 33 kDa mass (Fig. 2a, denoted with star), which approximates the calculated molecular weight of CXXC5 along with a number of proteins with varying molecular masses (Fig. 2a), as was also observed with cells transfected with an expression vector bearing no cDNA (Vector). To better evaluate the electrophoretic mobility of the endogenous CXXC5, we transiently transfected MCF7 cells with an expression vector bearing none (Vector), wild-type CXXC5 (WT-CXXC5) or the Flag-tagged cDNA (Flag-CXXC5), the latter which bears sequences encode for the Flag epitope at the amino-terminus of the resulting protein. We found that the overexpressed WT-CXXC5 or Flag-CXXC5 is detected with ab106533 at comparable levels primarily in the nuclear fraction (Supplemental data, Fig. 1), which approximates the estimated molecular mass of CXXC5 in vector transfected cells (Fig. 2a). To ensure the identity of the endogenous CXXC5 protein, we used an siRNA approach to reduce/ablate the intracellular levels of the endogenous protein. For this purpose, we utilized the FlexitubeGene Solution (Qiagen, Germany) that contains four siRNAs (the siRNA sequences were Supplemental Data, Table 1), three of which specifically target encoding *CXXC5* transcript (siRNA #2, 7 and 10) and one (siRNA #9) targets 3′UTR of the transcript. We also used AllStars Negative Control siRNA (NC, Qiagen, Germany) as negative control. To confirm that siRNAs indeed reduce the intracellular levels of the CXXC5 transcript, we initially subjected total RNA samples from MCF7 cells transfected with a CXXC5 specific siRNAs or AllStars NC siRNA to RT-qPCR. For the normalization of results, we used the expression of *RPLP0*. We found that siRNA#2 or siRNA#10 effectively repressed, whereas siRNA#7 or siRNA#9 had minimal effects on, the intracellular levels of the *CXXC5* transcript compared to levels observed in un-transfected or NC transfected cells (Fig. 2b). We then transfected MCF7 cells with NC, siRNA#2, #9 and #10 or without and with an expression vector bearing none or WT-CXXC5 cDNA as control. The nuclear fraction of transfected cells was subjected to WB (Fig. 2c). Result revealed that siRNA#10 effectively repressed, siRNA#2 and siRNA#9 reduced, the amount of a specific protein with a molecular mass similar to that of CXXC5 without an effect on other proteins detectable by the antibody. Based on these results, we inferred that the protein with an electrophoretic migration approximating 33 kDa molecular mass is the endogenous CXXC5 protein.

It is also evident that the endogenous CXXC5 protein shows a faster electrophoretic migration compared to the overexpressed WT-CXXC5 or Flag-CXXC5, denoted with an arrow. While the migratory difference between Flag-CXXC5 and WT-CXXC5 is likely due to the Flag epitope (approximately 1013 Da) present at the amino-terminus of the Flag-CXXC5 in WB, the reason of the migratory difference between the endogenous CXXC5 and exogenously introduced WT-CXXC5 is not clear. The amount and/or aberrant post-translational modifications of overexpressed CXXC5 proteins could have affected the electrophoretic migration compared to the endogenous protein.

To examine the effect of E2 on CXXC5 protein levels, we treated MCF7 cells, grown in the presence of CD-FBS for 48 h, without or with 10^−9^ M E2 and/or 10^−7^ M ICI for 24 h. The nuclear extracts were then subjected to WB (Fig. 2d). As observed with the *CXXC5* transcript, quantitative analysis revealed that E2 augmented the level of CXXC5 protein compared to that of vehicle control (EtOH, 0.01%). ICI alone had no effect on CXXC5, while it effectively prevented the E2-mediated increase in protein levels. Treatments, as expected, had no effect on HDAC1, histone deacetylase 1, whose levels were assessed as control. Thus, our results indicate that the enhanced transcription of *CXXC5* by E2-ERα is reflected in an increase in the level of CXXC5 protein.

### CXXC5 is localized primarily in the nucleus of MCF7 cells

**Figure 3:**
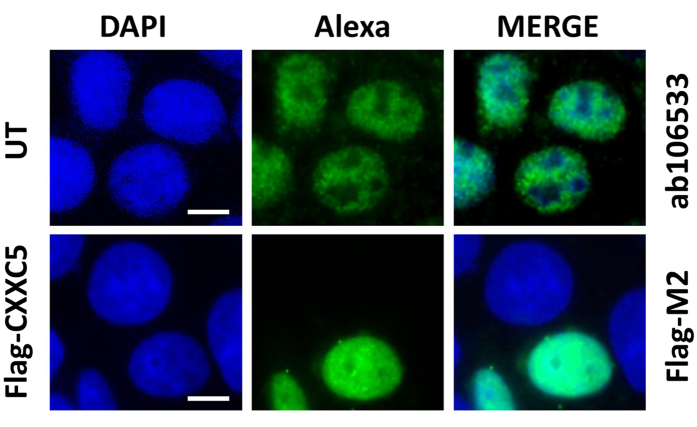
Intracellular Localization of CXXC5. MCF7 cells grown on coverslips in 12-well culture plates with medium containing FBS for 48 h were un-transfected (UT) or transfected with pcDNA3.1(−) bearing the Flag-CXXC5 (F-C5) cDNA. Thirty six hour after, cells were fixed with 2% paraformaldehyde in PBS and permeabilized with 0.4% Triton-X100 in PBS. For the detection of the endogenous CXXC5 protein in un-transfected cells, cells were blocked with 10% normal goat serum (NGS) followed by an incubation with ab106533 in PBS containing 2% NGS. Cells were then incubated with an Alexa Fluor^®^-488 (green channel) conjugated goat anti-rabbit secondary antibody in PBS containing 2% NGS to detect endogenous CXXC5. For the detection of Flag-CXXC5 protein in transfected cells, following a block with 10% bovine serum albumin (BSA) in PBS, cells were incubated with the Flag-M2 antibody in PBS containing 3% BSA. Cells were then incubated with an Alexa Fluor^®^-488 (green channel) conjugated goat anti-mouse secondary antibody in PBS containing 3% BSA. Nuclei were stained with 4′,6-diamidino-2-phenylindole (DAPI). (blue channel). Merge images are indicated. A representative image from two independent experiments is shown. Scale bar is 5 μm.

CXXC5 contains a nuclear localization signal and was shown to reside in the cytosol or the nucleus depending on particular cell type in different tissues^12^,^13^,^14^. A previous study using MCF7 cells transiently transfected with an expression vector bearing Flag-CXXC5 cDNA located Flag-CXXC5 in the nucleus^12^. Our result that CXXC5 is primarily detected in the nuclear extract also suggests that CXXC5 is a nuclear protein in MCF7 cells. To examine this issue, we carried out immunocytochemistry (ICC) using the ab106533 or the Flag-M2 antibody with un-transfected cells for the endogenous CXXC5 localization and with cells transiently transfected with an expression vector bearing the Flag-CXXC5 cDNA. The ab106533 antibody detected CXXC5 in the nuclei of the un-transfected (Fig. 3, UT) or transfected cells with an expression vector bearing no cDNA (data not shown). Similarly, the overexpressed Flag-CXXC5 was detected in the nucleus with the Flag-M2 antibody (Fig. 3, Flag-CXXC5). The nuclear staining is specific to the Flag-CXXC5 protein, as we observed no staining with Flag-M2 in some cells of the transiently transfected cell population. These results therefore indicate that CXXC5 is a nuclear protein in MCF7 cells.

### The binding of ERα to an ERE sequence in the *CXXC5* gene locus

To begin to address how E2-ERα regulates the expression of *CXXC5*, we subjected the sequences of the *CXXC5* gene locus to the Dragon ERE finder version 3 (System for Identification and Interactive Analyses of Estrogen Response Elements in DNA Sequences; http://datam.i2r.a-star.edu.sg/ereV3/). We used a stringent analysis with a matrix of maximum one mismatch in the sequence compared to the core consensus ERE (GGTCAnnnTGACC) based on our earlier findings that one mismatch in the core consensus sequence is tolerated to allow ERα interaction with ERE^7^. Our *in silico* analysis revealed a putative ERE sequence (GGTCAggaTGACA; wherein the non-consensus nucleotide A is underlined) starting at position −242 from the translation initiation codon (ATG, A being +1; Supplementary data, Fig. 2).

**Figure 4:**
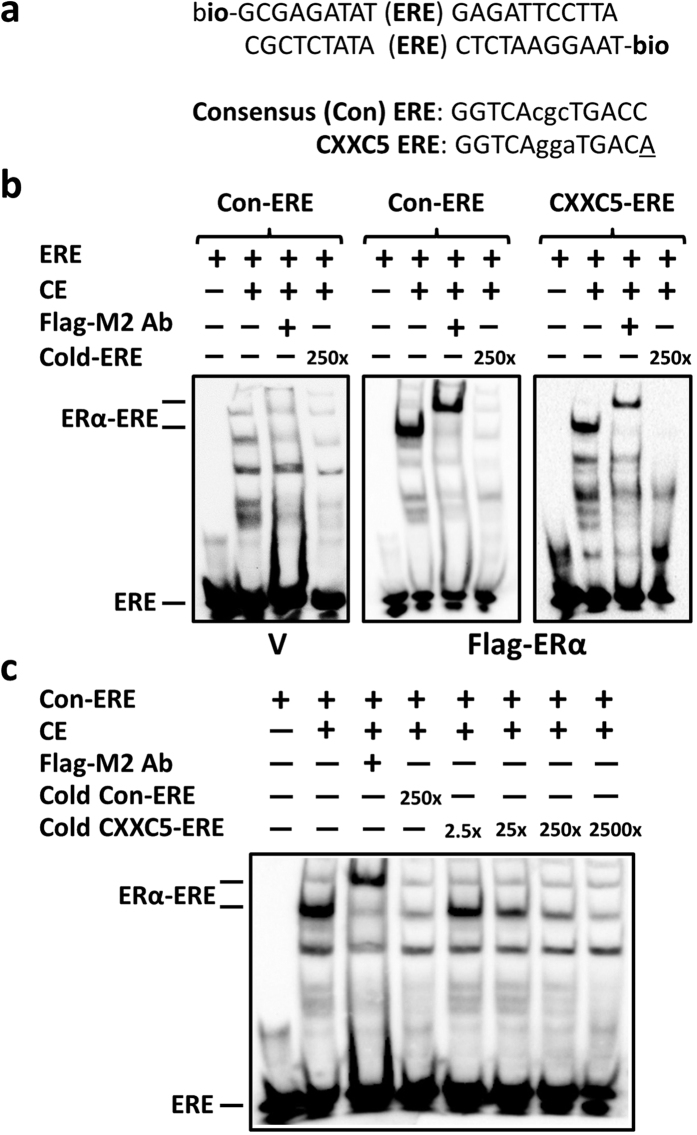
Electrophoretic mobility Shift Assay (EMSA). (**a**) The upper and lower oligomer sequences, which surround (parenthesis) the consensus-ERE (Con-ERE) or CXXC5-ERE test sequence, are biotinylated at 5′-ends. Underlined A residue in CXXC5-ERE indicates the variation from the consensus. (**b,c**) Cell extracts (CE; 10 μg) of MDAMB231 cells transfected with pcDNA3.1(−) bearing none (Vector, V) or the Flag-ERα cDNA were subjected to EMSA using biotinylated DNA (40 fmol) with (+) or without (−) the Flag-M2 antibody (Flag-M2) in the absence (−) or presence (+) of cold competitor at indicated amounts. ERα-ERE denotes the protein-bound biotinylated ERE. ERE indicates the unbound (free) biotinylated ERE. A representative result from three independent determinations is shown.

To test whether ERα interacts with this putative ERE sequence of *CXXC5 in vitro*, we utilized electrophoretic mobility shift assay (EMSA) using DNA substrates conjugated with biotin at 5′ ends. The DNA substrate consists of a 13 bp core consensus (Con)-ERE or CXXC5-ERE sequence embedded (parenthesis) within a larger oligomer with no ERE features to ensure the specificity of ER-ERE interactions (Fig. 4a) 7. We then assessed the binding ability of ERα to ERE using extracts of MDAMB231 cells transiently transfected with an expression vector bearing none as control (V) or Flag-ERα cDNA by EMSA, as we described previously^7^. Cellular extracts transfected with an expression vector bearing the Flag-ERα cDNA, but not with the parent vector (V), shown in the presence of the consensus ERE containing DNA, displayed an interaction with DNA bearing the consensus-ERE (Con-ERE) or the CXXC5-ERE (Fig. 4b). The presence of the Flag-M2 antibody in the reaction further retarded electrophoretic migration of ERα-ERE complexes, emphasizing the specificity of protein-DNA interaction. The inclusion of a 250-fold molar excess of the un-labeled (cold) ERE prevented the complex formation between ERα and the biotin-labeled ERE. Moreover, the CXXC5-ERE effectively competed with the consensus ERE as the cold CXXC5-ERE at increasing concentrations diminished the ERα-consensus ERE complex (Fig. 4c). These results, together with our observations that a DNA fragment bearing no-ERE features did not affect the binding of ERα to CXXC5-ERE in EMSA (Supplementary Data, Fig. 3), indicate that ERα specifically interacts with the CXXC5-ERE sequence *in vitro*.

**Figure 5:**
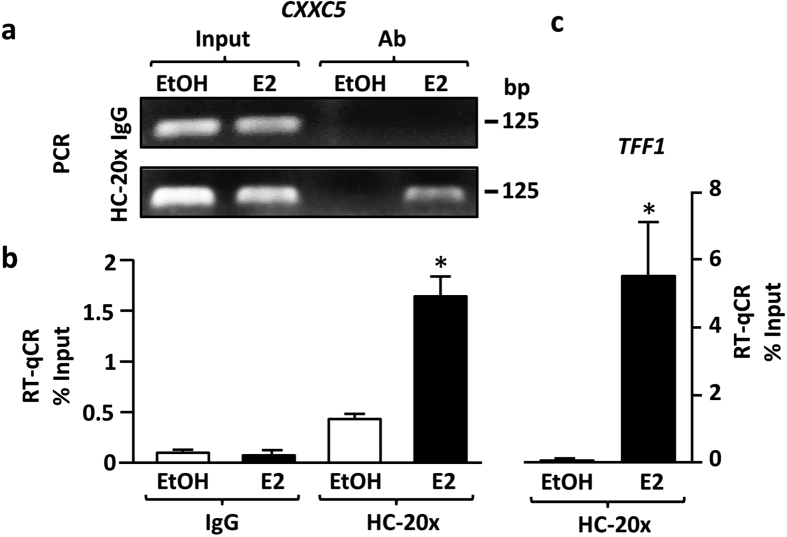
Chromatin Immunoprecipitation assay (ChIP). MCF7 cells grown in medium containing CD-FBS for 72 h treated without (EtOH, 0.01%) with 10^−8^ M E2 for 1 h prior to ChIP. Cells were fixed with 0.75% paraformaldehyde, lysed, sonicated and subjected to ChIP using IgG or an ERα specific HC20x antibody followed by the incubation with Protein A/G conjugated magnetic beads. Shown (**a**) are PCR reactions subjected to 2% agarose gel electrophoresis from a representative experiment performed three independent times. (**b**) Samples were also subjected to RT-qPCR for quantitative analysis with primers specific to the estrogen responsive region of *CXXC5*. (**c**) RT-qPCR results the estrogen responsive region of *TFF1* with the same experimental inputs described in **(b)** with primers specific to the estrogen responsive region of *TFF1*. Sizes of the DNA fragments in base pairs are indicated. Asterisk (*) denotes significant change depicted as percent (%) of input.

To ensure that ERα also binds to the putative ERE sequence of the *CXXC5* gene in cellular context, we carried out chromatin immunoprecipitation assay (ChIP), as described previously^26^. MCF7 cells grown for 72 h in the absence of steroid hormones were treated with EtOH (0.01%) or E2 at a saturating concentration, 10^−8^ M, for 1 h. Cells were then cross-linked and lysed. After DNA shearing, cell extracts were subjected to immunoprecipitation using a ChIP-grade ERα-specific HC-20x antibody. Following crosslinking reversal and protein digestion, PCR and RT-qPCR were carried out using isolated DNA samples. Qualitative PCR results revealed that the E2 treatment augments the interaction of ERα with the ERE sequence of *CXXC5* positioned residues between −242 through −255 (Fig. 5a). RT-qPCR results further revealed that E2 quantitatively augments the binding of ERα to the ERE sequence of *CXXC5* (Fig. 5b). Similarly, RT-qPCR showed that E2 enhances the binding of ERα to the ERE of the *TFF1* gene (Fig. 5c), a non-consensus ERE sequence that mediates the E2-ER responsiveness of the gene^26^,^27^, as we showed previously^26^,^28^. These results indicate that ERα in the presence of E2 effectively interacts *in cellula* with the ERE sequence of the *CXXC5* gene.

### The effect of E2-ERα on the estrogen responsive region of *CXXC5*-driven reporter enzyme activity

**Figure 6:**
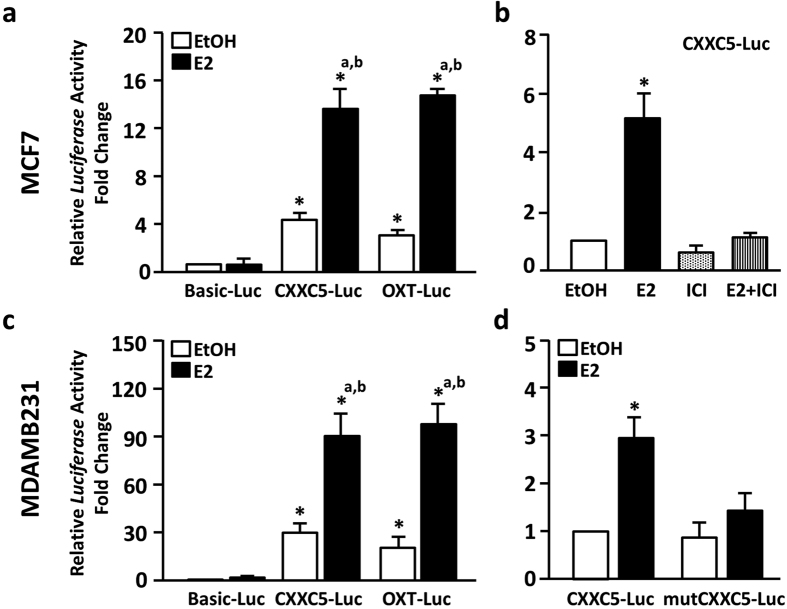
Transcriptional responses from the estrogen responsive *CXXC5* region-driven gene reporter. (**a**) MCF7 cells grown in medium containing CD-FBS for 48 h were transiently transfected with pGL3 bearing none (Basic-Luc), the estrogen responsive region of *CXXC5* (CXXC5-Luc) or *OXT* (OXT-Luc) driving *Firefly Luciferase* cDNA expression as the reporter in the absence (EtOH, 0.01%) or presence of 10^−9^ M E2 for 24 h. The transfection efficiency was monitored by the co-expression of pCMV–RL that drives the expression of *Renilla Luciferase* cDNA. 24 h later, cellular extracts were subjected to luciferase assays. Shown is the mean ± SD of three independent experiments performed in triplicate. *Firefly/Renilla* luciferase activities are presented as fold change compared to EtOH control of pGL3-Basic, which is set to 1. *^a^ and *^b^ indicate significant difference from E2 of Basic-Luc and the corresponding EtOH control, respectively. (**b**) MCF7 cells transfected with CXXC5-Luc treated without (EtOH, 0.01%) or with 10^−9^ M E2 and/or 10^−7^ M ICI for 24 h were subjected to luciferase assays. Shown is the mean ± SD of three independent experiments performed in triplicate. *Firefly/Renilla* luciferase activities are presented as fold changes compared to EtOH, which was set to 1. (**c**) MDAMB231 cells were transfected as described in (A) with Basic-Luc, CXXC5-Luc, or OXT-Luc reporter together with pCDNA-Flag-ERα vector. Cells were also co-transfected with pCMV-RL for monitoring transfection efficiency. Results are the mean ± SD of three independent experiments performed in triplicate. *Firefly/Renilla* luciferase activities are presented as fold changes compared to EtOH of pGL3-Basic, which is set to 1. *^a^ indicate significant change from EtOH of Basic-Luc; while *^b^ denotes significant change of E2 compared to EtOH of CXXC5-Luc or OXT-Luc. (**d**) MDAMB231 cells were transfected with CXXC5-Luc or mutCXXC5-Luc vector, the latter which bears a mutant sequence that changes the ERE sequence in *CXXC5* to a non-ERE, together with pcDNA-Flag-ERα vector. Cells were treated without (EtOH, 0.01%) or with 10^−9^ M E2 for 24 h. Shown is the mean ± SD of three independent experiments performed in triplicate. The normalized *Firefly/Renilla* luciferase activities are presented as fold change compared to EtOH of CXXC5-Luc, which was set to 1.

To examine the region of *CXXC5* containing the ERE sequence is indeed responsive to ERα when E2 is present, we generated a reporter vector (CXXC5-Luc) bearing a 305 bp PCR fragment (−305 to +1, +1 being the A residue in the encoding ATG codon) of the *CXXC5* gene from the genomic DNA of MCF7 cells as template. The vector bears the *Firefly Luciferase* cDNA as the reporter enzyme. MCF7 cells grown in CD-FBS containing medium for 48 h were transfected with the reporter vector bearing none (Basic-Luc) or the ERE containing E2 responsive region of *CXXC5* (CXXC5-Luc) without (EtOH, 0.01%) or with 10^−9^ M E2 and/or 10^−7^ M ICI for 24 h. Cells were also transfected, as control, with the *Oxytocin* (OXT-Luc) reporter vector which confers E2-ERα responsiveness through an ERE, as described previously^7^,^29^. In MCF7 cells, the basal reporter activity (in the presence of EtOH) of the CXXC5-Luc and OXT-Luc was significantly higher compared to the Basic-Luc. E2 treatment had no effect on luciferase enzyme levels from the Basic-Luc. E2, on the other hand, augmented the reporter enzyme activity from the CXXC5-Luc or OXT-Luc construct compared to EtOH treated Basic-Luc and the corresponding EtOH control (Fig. 6a). That ICI effectively blocked the increase in luciferase levels in response to E2 also suggests that E2-mediated responses from the CXXC5 (or OXT, data not shown) construct is due to ERα (Fig. 6b). Indeed, in MDAMB231 cells transiently transfected with expression vector bearing the Flag-ERα cDNA, but not from the construct bearing no cDNA (data not shown), together with the reporter constructs, E2 when ERα is present augmented the reporter enzyme activity from the CXXC5-Luc or OXT-Luc construct without affecting the enzyme activity from Basic-Luc (Fig. 6c). Moreover, the E2-ERα responsiveness of the *CXXC5* construct is due to the presence of the ERE sequence. This is because, a CXXC5 reporter construct bearing mutations only in the ERE sequence, which changes 5′- GGTCAggaTGACA-3′ to 5′-TTTGATCCCTCAA-3′ by overlapping PCR, lost its responsiveness to E2-ERα (Fig. 6d). Thus, these results collectively indicate that E2-ERα induces the transcription of *CXXC5* by interacting with ERE.

## Discussion

The identification of estrogen target genes and the elucidation of mechanisms responsible gene expressions have critical importance in defining the regulatory network of E2-ERα actions in target tissue physiology and pathophysiology. In assessing the mechanism by which E2-ERα mediates the CXXC5 gene expression in a cell model derived from breast adenocarcinoma, we show here that E2-ERα regulates the transcription of CXXC5 by a direct interaction with an ERE sequence present at a region upstream of the initial translation codon of the *CXXC5* locus.

The accumulating evidence suggests that CXXC5 as a protein partner, transcription factor and/or epigenetic modulator is involved in cellular proliferation, differentiation and death in response to various signaling in diverse tissues and organs^30^. Identified as a novel, all-trans retinoic acid (ATRA)-responsive gene, the CXXC5 protein was shown to be involved in ATRA-induced terminal differentiation of myelocytic leukemia cells, and in cytokine-driven physiological myelopoiesis^12^. CXXC5 as a transcription factor appears also to be critical for bone morphogenic protein 4 (BMP4)-induced endothelial cells differentiation and myogenesis^31^, Wilms tumor 1 (WT1)-mediated nephrogenesis^14^ as well as Wnt/β-catenin signaling pathway-regulated oligodendrocyte differentiation and myelination^32^. Moreover, it was also suggested that CXXC5 acts as a transcription repressor for hypoxia-mediated Cytochrome C oxidase subunit 4/2 (*COX4/2*) through a direct interaction with an oxygen responsive element in the proximal promoter of the gene^16^. Interestingly, CXXC5 was reported to act as a vitamin D (VitD) receptor interactor to enhance or repress transcription of VitD responsive genes depending on the corresponding promoter components^21^.

Apart from being a transcription factor, CXXC5 was shown to act as a negative-feedback regulator of Wnt/β-catenin signaling by interacting with the cytoplasmic scaffold Dishevelled (Dvl) proteins in osteoblast differentiation and bone formation^33^, cutaneous wound healing and collagen production^34^, neural stem cell differentiation and telencephalon development^13^. CXXC5 was also reported to interact with and require for DNA damage-induced ATM phosphorylation, subsequent activation of p53, cell cycle arrest and apoptosis^35^. Interactions of CXXC5 with SMAD proteins was suggested to be critical for Tumor Necrosis Factor α (TNFα)-induced apoptosis as well^36^.

Moreover, it was recently reported that CXXC5 is a key molecule to repress CD40L in CD8+ cytotoxic T cells through epigenetic regulation^37^. It appears in a murine model that Cxxc5, which is repressed by Th-inducing pox virus and zinc finger/Kruppel-like factor (ThPOK), inhibits CD40L expression and induces the methylation of H3K9 in the promoter region of the Cd40lg gene through an interaction with Suppressor of Variegation 3–9 Homolog 1 (SUV39H1), a histone-lysine methyltransferase^37^.

Corroborating the importance of CXXC5 in cellular events, de-regulation of *CXXC5* expression appears to correlate with a number of pathologies including diminished ovarian reserve (DOR), Blepharophimosis Ptosis Epicantus inversus Syndrome (BPES), cardiovascular disease, myelodysplastic syndrome^19^,^20^,^21^,^22^. Altered CXXC5 expression was also found to be associated with locally advanced breast tumors, metastatic malignant melanomas, papillary thyroid carcinomas^18^ and Acute Myeloid Leukemia^38^.

Despite the emerging importance of CXXC5 in physiology and pathophysiology of various organ and tissues, the mechanism by which the expression of *CXXC5* expression is regulated in response to signaling pathways or the regulatory regions critical for *CXXC5* transcription remains largely unexplored. Based on an *in silico* analysis, the responsiveness of *CXXC5* to ATRA was suggested to be regulated through a retinoid-response element present at a region upstream of the transcription start site^12^. Experimentally, WT1 was shown to activate the transcription of *CXXC5* by a direct interaction with DNA sequences at an upstream enhancer region of the gene^14^. On the other hand, ThPOK-mediated transcriptional repression of the mouse *Cxxc5* appears to be dependent upon several regions in the first intron of the *Cxxc5* gene^37^. These, together with our demonstration here that ERα regulates the transcription of *CXXC5* by a direct interaction with an ERE sequence present at a region upstream of the initial translation codon of *CXXC5* by EMSA, ChIP and reporter assays suggest that integrated effects of transcription factors acting synergistically and/or antagonistically in response to various signaling pathways are ultimately responsible for the expression of *CXXC5* through interactions with regions in the *CXXC5* locus. Interestingly, genes for both retinoic acid receptor α, *RARA*, which acts as a receptor for ATRA^39^, and *WT1*^10^ are E2 and ERα responsive. Moreover, RARα was shown to cooperate with ERα to regulate the expression of a set of estrogen responsive genes^40^. Observations that WT1 alters the expression of *ESR1* encoding ERα^41^ and interacts with ERα^42^ suggest a reciprocity between ERα and WT1 actions as well. It is therefore tempting to speculate that E2-ERα, in addition to a direct effect on *CXXC5* expression, also modulates the transcriptional output of CXXC5 by regulating gene expressions of and interactions with RARα and WT1 in breast tissue and breast adenocarcinomas.

One of our intriguing observations is that E2-mediated transcription of *CXXC5* occurs in a bi-phasic fashion such that the addition of E2 enhanced the expression of CXXC5 at 3 h and 24 h without an effect at 6 h in contrast to the *TFF1/pS2* expression, of which E2 augmented the transcription at all-time points tested. This could imply that the regulation of the *CXXC5* expression by E2-ERα is cell-cycle dependent. Indeed, our ongoing studies are revealing that this might be the case and also suggesting that CXXC5 shows an altered intra-nuclear distribution depending upon cell cycle phases, an event that could be associated with cycle-dependent functions of the protein. A cell-cycle dependent re-modelling of CXXC5 chromatin locus to a non-permissive state for E2-ER signaling could also underlie the bi-phasic effect of E2-ERα on *CXXC5* expression.

The expression of E2 responsive primary genes mediated by E2-ER encompasses proteins involved in the metabolism of nucleic acid/proteins, transcription factors, membrane signaling cascade and receptor proteins^9^,^10^,^43^,^44^,^45^. These proteins in turn participate in the regulation of secondary gene expressions responsible for DNA repair and cell cycle progression and, consequently, in the initiation of E2-mediated cellular proliferation^9^,^10^,^43^,^44^,^45^. Synthesized as the primary response gene product in response to E2-ERα signaling and acting as a protein partner, transcription factor and/or epigenetic modulator in a cell-cycle dependent manner CXXC5 could participate in the regulation of secondary gene expressions responsible for cellular division. CXXC5 could concomitantly involve in the suppression of transcription of genes involved in cellular death. These integrated events may be critical for the ability of E2-ERα to induce cellular proliferation in breast tissue. One projection would then be that a de-regulated CXXC5 expression contributes to the initiation and/or progression of breast cancer. In keeping with this prediction is the findings using expression analyses of breast tumor and breast tumor data sets that high levels of CXXC5 expression is associated with poor prognosis and is an unfavorable prognostic factor in breast cancer without or with ER antagonist treatment^18^. Our ongoing studies aiming at the dissection of the role of CXXC5 in E2-ERα signaling could provide important insights into the mechanism of E2-mediated cellular events, and consequently the development of additional and/or alternative treatment modalities to combat breast cancer.

In conclusion, we show here that *CXXC5* is a *bona fide* E2-ERα responsive gene regulated through the ERE-dependent signaling pathway.

## Materials and Methods

### Plasmids

The human wild-type (WT) CXXC5 cDNA containing pMigR1 vector was kindly provided by Dr. Frederic Pendino, INSERM UMRS-1007, Paris, France. The human *CXXC5* gene encodes 322-amino acid-long protein. WT-CXXC5 cDNA with or without Flag epitope were obtained by PCR using CXXC5 specific cloning primers. The cDNA was then subcloned into pBS-KS (−) (Agilent, Santa Clara, CA, USA) and sequenced to ensure the fidelity of the encoding sequences. In the CXXC5 cDNA, the first methionine and the stop codon, which are underlined, are within the context of Kozak sequence (CGCCATG) and a PolyA (TAATAAA) signal to ensure efficient translation and translation termination, respectively. The CXXC5 cDNA was then transferred into the mammalian expression vector pcDNA3.1 (−) (Thermo-Fisher Scientific Inc., Waltham, MA, USA) with appropriate restriction enzymes. The human ERα cDNA containing sequences encoding the amino-terminally located Flag epitope was described previously^46^.

For reporter assays, we used pGL3-Basic Luciferase Reporter vector that bears the *Firefly Luciferase* cDNA as the reporter enzyme (Promega Corp., Madison, WI, USA). The reporter plasmid bearing the estrogen responsive region of *Oxytocin* (OXT) that contains a non-consensus ERE was described previously^29^. In this OXT reporter vector, a 334-bp fragment (−334 to +1, +1 being the A residue in the translation initiation codon, ATG) drives the expression of the *Firefly Luciferase* enzyme cDNA. For the engineering of the reporter vector bearing the estrogen responsive CXXC5 region a DNA fragment of 305 bp (−305 to +1, +1 denotes the A residue in the first encoding ATG of *CXXC5*) generated by PCR using the genomic DNA of MCF7 cells as template (Supplemental Data, Fig. 2) was inserted into pGL3-Basic with appropriate restriction enzymes. We used an overlapping PCR approach using pGL3 vector bearing the estrogen responsive region of *CXXC5* as template to generate a mutant CXXC5 region (mutCXXC5). In this mutant, 5′-GGTCAggaTGACA-3′ sequence is converted to a non-ERE sequence, 5′-TTTGATCCCTCAA-3′. The resultant DNA fragment was then inserted into pGL3-Basic vector with appropriate restriction enzymes and sequenced. In transfections, transfection efficiency was monitored with a reporter vector bearing CMV promoter that drive the expression of the *Renilla Luciferase* cDNA (pCMV-RL, Promega), which we described previously^46^,^47^. Luciferase assays were performed with a Dual Luciferase Assay kit (Promega) according to the manufacture′s recommendations.

Restriction and DNA modifying enzymes were obtained from New England Bio-Labs (Beverly, MA, USA). 17β-estradiol (E2) was purchased from Sigma-Aldrich (St. Louis, MO, USA). The Flag antibody (Flag-M2) was purchased from Sigma-Aldrich. The antibodies for β-actin (ab8227), HDAC1 (ab19845) and CXXC5 (ab106533) were purchased from Abcam Inc. (Cambridge, MA, USA). An ERα specific antibody (HC-20x) was obtained from Santa Cruz Biotechnologies (Santa Cruz, CA, USA). The complete antagonist of ER Imperial Chemical Industries 182,780 (ICI) was purchased from Tocris Biosciences (Ellisville, IL, USA). Secondary antibodies conjugated with horse radish peroxidase were purchased from Santa Cruz Biotech. Secondary antibodies conjugated with Alexa Fluor^®^ were obtained from Abcam Inc. siRNAs for CXXC5 were purchased from Qiagen Inc. (Düsseldorf, Germany).

### Cell Culture and Transfections

Culturing of cells was carried out as described previously^46^,^47^,^48^,^49^. For RNA or protein isolation, MCF7 cells in six-well tissue culture plates were maintained for 48 h in medium containing 10% charcoal dextran-stripped fetal bovine serum (CD-FBS). Cells were then treated without (Ethanol, EtOH, 0.01%) or with 10^−9^ M E2 and maintained for 3, 6 and 24 hours. At the termination, cells were subjected to total RNA isolation (Miniprep RNA isolation kit, ZymoResearch, Irvine, CA, USA) or protein extraction (NE-PER protein extraction kit, Thermo-Fisher). RNA and protein contents were assessed with NanoDrop (Thermo-Fisher) and Bradford Protein Assay (Bio-Rad Life Sciences Inc., Hercules, CA, USA), respectively.

### siRNA Transfection

MCF7 cells in 12-well tissue culture plates for RT-qPCR or six-well tissue culture plates for western blot analysis were transiently transfected with HiPerfect transfection reagent (Qiagen) using 75 ng of a CXXC5 siRNA (FlexiTube GeneSolution GS51523, Qiagen). Twenty-four hour after transfection, cells were subjected to total RNA isolation (ZymoResearch) or protein extraction (Thermo-Fisher).

### PCR and RT-qPCR

Isolated total RNA from cells treated without or with ligand was used for the cDNA synthesis (The RevertAid First Strand cDNA Synthesis Kit, Thermo-Fisher). The SYBR^®^ Green Mastermix (Roche Applied Science, Indianapolis, IN, USA), *CXXC5* specific primers (Forward Primer, FP: 5′-CGGTGGACAAAAGCAACCCTAC-3′ and Reverse Primer, REP: 5′-CGCTTCAGCATCTCTGTGGACT-3′) or *TFF1* primers (FP: 5′-TTGTGGTTTTCCTGGTGTCA-3′ and REP: 5′-CCGAGCTCTGGGACTAATCA-3′) were used for RT-qPCR reactions. For the normalization of results, we used the expression of *RPLP0* (FP: 5′-GGAGAAACTGCTGCCTCATA-3′ and REP: 5′-GGAAAAAGGAGGTCTTCTCG-3′). The relative quantification of reaction efficiency was assessed with the comparative 2^−ΔΔC^_T_ method^50^. During the RT-qPCR experiments MIQE Guidelines were followed^51^.

### Western Blot (WB)

WB was carried out as described previously^46^,^47^,^49^. In brief, MCF7 cells grown in six-well tissue culture plates in medium supplemented with CD-FBS for 48 h were treated without (EtOH, 0.01%) or with 10^−9^ M E2 and/or 10^−7^ ICI for 3, 6 or 24 h. At the termination, cells were collected and protein isolation was performed using NE-PER protein extraction kit (Thermo-Fisher). Protein content in extracts was measured with Bradford Protein Assay (Bio-Rad). Nuclear extracts (25 μg or 100 μg) were then subjected to SDS 10%-PAGE. Proteins were probed with an antibody specific to CXXC5 (ab106533, Abcam) or Flag (Sigma-Aldrich) followed by a secondary antibody conjugated with the horseradish peroxidase (Santa Cruz). Protein images were developed using the ECL-Plus Western Blotting kit (GE Healthcare Bio-Sciences, Pittsburgh, PA, USA) and captured with ChemiDoc™ Imaging System (Bio-Rad). Precision Plus Protein™ Dual Color Standards (Bio-Rad) was used as molecular marker in WB. The quantification of images was carried out using ImageJ image processing program (https://imagej.nih.gov/ij/).

### Immunocytochemistry (ICC)

MCF7 cells were subjected to ICC as described previously^7^,^48^,^49^. In brief, cells grown on coverslips in 12-well tissue culture plates for 48 h were transiently transfected with TurboFect transfection reagent (Thermo-Fisher) using one μg mammalian expression vector pcDNA3.1 (−) bearing none (as control) or the Flag-CXXC5 cDNA for 36 h. Cells were then fixed by 2% paraformaldehyde and treated with 0.4% Triton-100X (Sigma-Aldrich) for permeabilization. Cells were blocked with 10% Bovine Serum Albumin (BSA) in PBS for Flag-M2 (Sigma Aldrich) or 10% Normal Goat Serum (NGS) for the ab1056533 antibody. AlexaFluor^®^ 488 conjugated goat anti-mouse (ab150113, Abcam) secondary antibody diluted in 3% BSA for Flag-M2; whereas AlexaFluor^®^ 488 conjugated goat anti-rabbit (ab150077, Abcam) secondary antibody was diluted in 2% NGS for ab106533. DAPI (4,6-diamido-2-phenylindole hydrochloride; Vectashield, Vector Laboratories, Inc., Burlingame, CA) was used for nucleus staining.

### *In silico* analysis for ERE sequence

The dragon ERE finder version 3, System for Identification and Interactive Analyses of Estrogen Response Elements in DNA Sequences^52^ was used for the prediction of potential ERE sequences in the *CXXC5* locus.

### Electrophoretic mobility Shift Assay (EMSA)

EMSA was carried as described previously^46^,^48^,^49^ with the exception that oligomers contain none or 5′ end biotin label. Oligomers bearing the consensus ERE or CXXC5-ERE sequences (Fig. 4a) were purchased from Integrated DNA Technologies (IDT; Coralville, IA, USA) and annealed. Double-stranded DNA fragments were incubated in the presence or absence of extracts (10 μg) of MDAMB231 cells transfected with expression vectors bearing none (control) or the Flag-ERα cDNA. Reactions were further incubated without or with the Flag-M2 antibody. Samples were subjected to electrophoresis on 5% non-denaturing polyacrylamide gel. The membrane was UV cross-linked, and the probes were visualized according to the LightShift Chemiluminescent EMSA (Thermo-Fisher) as instructed by the manufacturer. Images were captured with ChemiDoc™ Imaging System (Bio-Rad). The quantification of images was carried out using ImageJ image processing program (https://imagej.nih.gov/ij/).

### Chromatin Immunoprecipitation Assay (ChIP)

ChIP assays were carried out as described^26^,^48^. In brief, MCF7 cells grown in medium supplemented with CD-FBS in T75 tissue culture plates for 72 h were treated without or with 10^−9^ M E2 for 1 h. Cells were then fixed with 0,75% paraformaldehyde at room temperature for 10 min and lysed with Nuclei Lysis Buffer containing 1% SDS and sonicated. Cell debris was pelleted and supernatant was collected. After blocking, the supernatant was incubated with a ChIP specific ERα antibody (HC-20x; Santa Cruz Biotechnology Inc., Santa Cruz, CA, USA) or IgG (Santa Cruz) for overnight and subjected to precipitation with Protein A/G Magnetic Beads (New England BioLabs). After de-crosslinking and protein digestion, DNA was recovered with a PCR Cleaning Kit (Qiagen). Samples (2 or 4 μl of a 30 μl elution) were subjected to PCR and RT-qPCR using primers specific for the region containing ERE of *CXXC5*. RT-qPCR results were normalized using percent (%) of input approach^53^. For the RT-qPCR, *CXXC5* ChIP primers (Forward Primer, FP: 5′-AATGCCTGGTCAAGCACATG-3′ and Reverse Primer, REP: 5′-TCTTCACTCTGTCACAAGAGGA-3′) or *TFF1* ChIP primers (FP: 5′-CCTGTGGCCCAGCCACTGCGTCTTTCAG-3′ and REP: 5′-CCTATCTCCTTGGGAGAGCTGTGAG-3′) were used.

### Statistical Analysis

Results were presented as the mean ± standard deviation (SD). Significance was determined using a two-tailed unpaired t test with a confidence interval, minimum, of 95%.

## Additional Information

**How to cite this article**: Yaşar, P. *et al*. Estradiol-Estrogen Receptor a Mediates the Expression of the *CXXC5* Gene through the Estrogen Response Element-Dependent Signaling Pathway. *Sci. Rep.* **6**, 37808; doi: 10.1038/srep37808 (2016).

**Publisher's note:** Springer Nature remains neutral with regard to jurisdictional claims in published maps and institutional affiliations.

## Supplementary Material

Supplementary Information
