# Supplementary material for: Estradiol-Estrogen Receptor α Mediates the Expression of the CXXC5 Gene through the Estrogen Response Element-Dependent Signaling Pathway
Source: Sci Rep. 2016 Nov 25;6:37808. doi: 10.1038/srep37808 (PMC5122896; doi:10.1038/srep37808)
Supplement: Supplementary Information [file srep37808-s1.pdf]

Estradiol-Estrogen Receptor α Mediates the Expression of the CXXC5 Gene through the Estrogen Response Element-Dependent Signaling Pathway

Pelin Yaşar, Gamze Ayaz and Mesut Muyan

SUPPLEMENTARY INFORMATION

SUPPLEMENTAL DATA

Fig. 1. Assessment of the endogenous CXXC5 protein in MCF7 cells. (**a**) Overexpressed CXXC5 protein overshadows the endogenous CXXC5. MCF7 cells were transfected with pcDNA3.1(-) bearing none (Vector, V) or the Flag-CXXC5 (F-C5) cDNA. Twenty-four hour after transfections, cells were subjected to protein extraction. Equal amounts (100 µg) of nuclear extracts of the parent vector (V) or Flag-CXXC5 cDNA transfected cells were run onto SDS 10%-PAGE and subjected to WB using a CXXC5-specific antibody, ab106533. Star in the vector transfected (V) lane denotes the putative endogenous CXXC5 protein, while arrows indicate the overexpressed Flag-CXXC protein in corresponding lane. Molecular weight marker is shown in kDa. A representative image from two independent experiments is shown. (**b & c & d**) MCF7 cells were transfected as indicated. Twenty-four hour after transfection cells were lysed and separated into nuclear and cytoplasmic fractions. To prevent the overshadowing effect of the overexpressed WT-CXXC5 or Flag-CXXC5 protein at 100 µg, 25 ug protein from the nuclear (**b**) and cytoplasmic (**c**) extract of the overexpressed WT-CXXC5 or Flag-CXXC5 protein as well as 100 µg of protein extracts from Vector (V) transfected cells were subjected to WB using ab106533. Star denotes the endogenous CXXC5 protein, while arrows indicate the overexpressed CXXC proteins. Molecular weight marker is shown in kDa. A representative image from two independent experiments is shown. (**d**) Overexposed image, six second exposure, of the WB image shown in B, which was exposed for two seconds. In all panels, β-Actin and HDAC1 were used as loading controls for cytoplasmic and nuclear extracts; the presence, albeit low amounts, of β-Actin in the nuclear extract and HDAC1 in the cytoplasmic extract indicates somewhat cross-contamination of cellular fractions. This suggests also that the detection of CXXC5 proteins in the cytoplasmic extracts is likely due to the contaminating nuclear extract.

Fig. 2. Nucleotide sequences of the estrogen responsive region of CXXC5. The 305 base-pair long *CXXC5* locus containing an ERE sequence was obtained by PCR using the genomic DNA of MCF7 cell and sequenced. The sequence (query) was then compared to sequences in human genome using Basic Local Alignment Search Tool (http://blast.ncbi.nlm.nih.gov/Blast.cgi). The ERE sequence (underlined in red) resides between residues -242 through -255 from the first encoding ATG (A being +1) codon of *CXXC5*, underlined in black. The PCR generated DNA fragment was inserted into the pGL3-Basic vector for the construction of the pGL3-CXXC5 reporter plasmid.

Fig. 3. Electrophoretic Mobility Shift Assay (EMSA). (**a**) Cell extracts (CE; 10 µg) of MDAMB231 cells transfected with pcDNA3.1(-) bearing none (Vector, V) or the Flag-ERα cDNA were subjected to EMSA using biotinylated DNA (40 fmol) with (+) or without (-) the Flag-M2 antibody (Flag-M2) in the absence (-) or presence (+) of cold competitor at indicated amounts. ERα-ERE denotes the protein-bound biotinylated ERE. ERE indicates the unbound (free) biotinylated ERE. (**b**) MDAMB231 cells were transfected with pcDNA3.1(-) bearing none (V) or the Flag-ERα cDNA. Cell extracts were subjected to EMSA using biotinylated CXXC5-ERE (40 fmol). Specific binding of ERα to CXXC5-ERE was examined in the presence (+) of excess (2500 fold) cold (unlabeled) DNA fragment with no-ERE features (5'-CTGTCAGTATCAGTTACAGGTCTA-3', upper stand).

Table 1. Nucleotide sequences of siRNAs. Shown are the nucleotide sequences of siRNAs used in experiments to assess the identity of the endogenous CXXC5 protein.

Supplemental Data, Figure 1

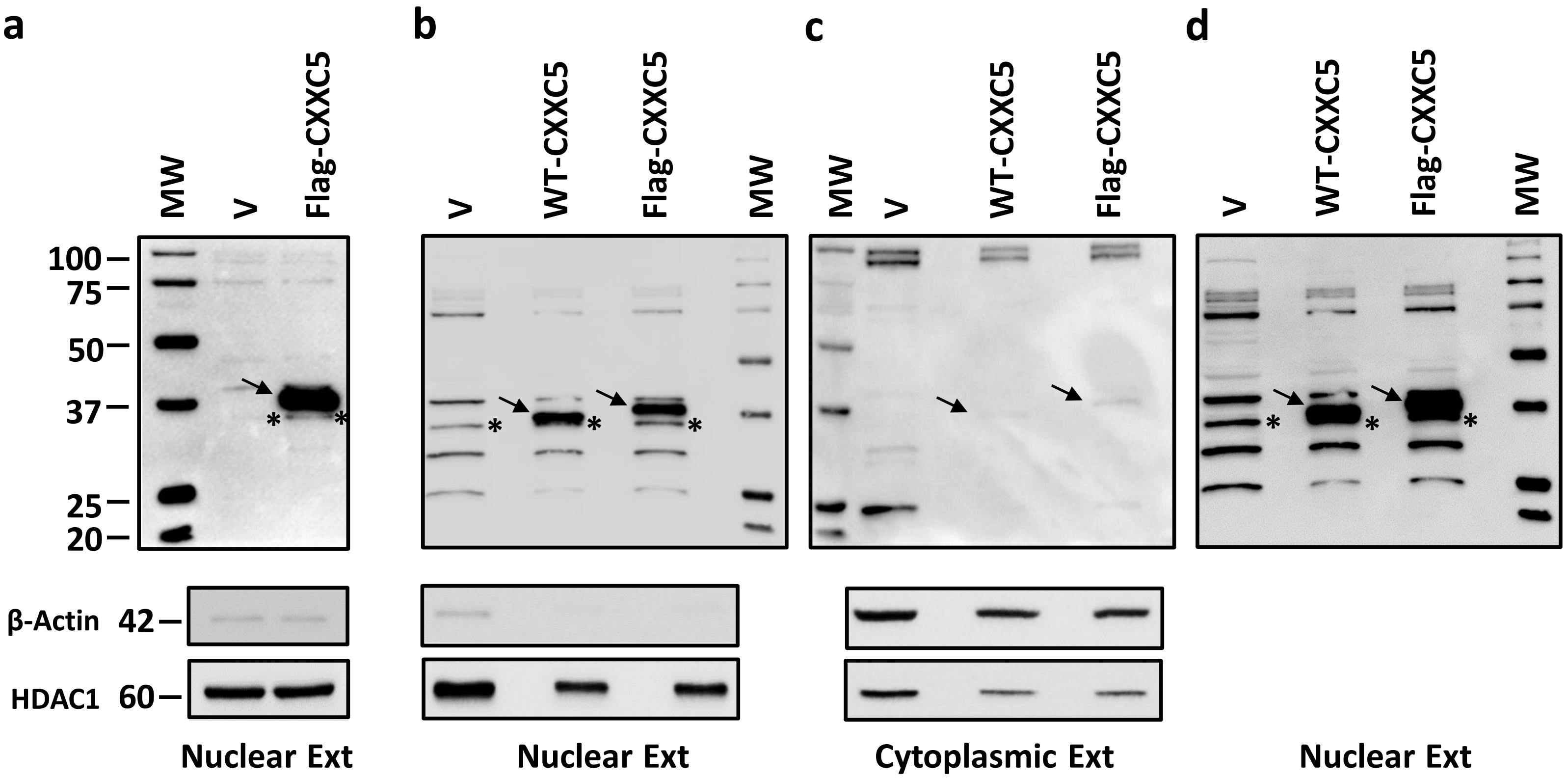

Supplemental Data, Figure 2

Homo sapiens chromosome 5, alternate assembly CHM1_1.1

Sequence ID: ref|NC_018916.2| **Length:** 180347728 **Number of Matches:** 1

Range 1: 138492850 to 138493157 GenBank Graphics ▼ Next Match ▲ Previous Match

Score	Expect	Identities	Gaps	Strand
569 bits(308)	5e-160	308/308(100%)	0/308(0%)	Plus/Plus

Features: CXXC-type zinc finger protein 5
CXXC-type zinc finger protein 5

```
                                                                          -255
Query  1          CTCACCAGGATGGATCTGGTGCTTAATAAATGCCTGGTCAAGCACATGGTGGTCAGGATG  60
                  ||||||||||||||||||||||||||||||||||||||||||||||||||||||||||||
Sbjct  138492850  CTCACCAGGATGGATCTGGTGCTTAATAAATGCCTGGTCAAGCACATGGTGGTCAGGATG  138492909
                      -242
Query  61         ACAGGACCGTTGATAGTGGCGGTGGTGGCGATGTTGAAGGGGGAGGTGTTCACTGCTGCC  120
                  ||||||||||||||||||||||||||||||||||||||||||||||||||||||||||||
Sbjct  138492910  ACAGGACCGTTGATAGTGGCGGTGGTGGCGATGTTGAAGGGGGAGGTGTTCACTGCTGCC  138492969

Query  121        CTGACCCTGTATCCTCTTGTGACAGAGTGAAGACATTTCCACCTGGACACCTGACCATGT  180
                  ||||||||||||||||||||||||||||||||||||||||||||||||||||||||||||
Sbjct  138492970  CTGACCCTGTATCCTCTTGTGACAGAGTGAAGACATTTCCACCTGGACACCTGACCATGT  138493029

Query  181        GCCTGCCCTGAGCAGCGAGGCCCACCAGGCATCTCTGTTGTGGGCAGCAGGGCCAGGTCC  240
                  ||||||||||||||||||||||||||||||||||||||||||||||||||||||||||||
Sbjct  138493030  GCCTGCCCTGAGCAGCGAGGCCCACCAGGCATCTCTGTTGTGGGCAGCAGGGCCAGGTCC  138493089

Query  241        TGGTCTGTGGACCCTCGGCAGTTGGCAGGCTCCCTCTGCAGTGGGGTCTGGGCCTCGGCC  300
                  ||||||||||||||||||||||||||||||||||||||||||||||||||||||||||||
Sbjct  138493090  TGGTCTGTGGACCCTCGGCAGTTGGCAGGCTCCCTCTGCAGTGGGGTCTGGGCCTCGGCC  138493149
                       +1
Query  301        CCACCATG  308
                  ||||||||
Sbjct  138493150  CCACCATG  138493157
```

From: http://blast.ncbi.nlm.nih.gov/Blast.cgi

Supplemental Data, Figure 3

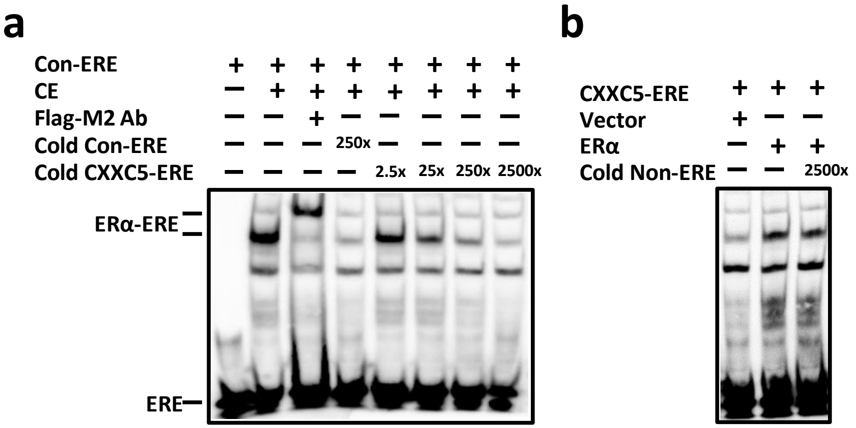

Supplemental Data, Table 1

siRNAs	siRNA sequence (5' to 3')	Target
Hs_CXXC5_2	CAGCAGTTGTAGGAATCGAAA	In the ORF of CXXC5
Hs_CXXC5_7	CAAGAGCGGTATCATCAGTGA	In the ORF of CXXC5
Hs_CXXC5_9	AGGGATTCGGGCGAAGACAAA	In the 3'UTR of CXXC5
Hs_CXXC5_10	TCAGATTTGCAAATTCAGAAA	In the ORF of CXXC5
AllStars Negative Control	Proprietary sequence	
